# 1374. Foodborne disease outbreak at a delivery pizza and sushi restaurant, Kazakhstan, 2023

**DOI:** 10.1093/ofid/ofad500.1211

**Published:** 2023-11-27

**Authors:** Feruza Ablimitova, Arystan Balmagambetov, Roberta Horth, Dilyara Nabirova

**Affiliations:** Central Asia FETP, Almaty, Almaty, Kazakhstan; Central Asia FETP, Almaty, Almaty, Kazakhstan; US Centers for Disease Control and Prevention, Dulles, Virginia; CDC Central Asia office, Almaty, Almaty, Kazakhstan

## Abstract

**Background:**

On February 3, 2023, the Kazakhstan Ministry of Health was notified of 71 hospitalized cases of acute gastointestinal illness among people who had ordered food from a delivery sushi and pizza restaurant in Talgar (population=50,000). We conducted an investigation from February 4 to 16 to identify risk factors.

**Methods:**

We conducted a retrospective cohort study among people who consumed food from the sushi bar from January 31 to February 1, 2023. To find additional cases, we used a line list of orders made by customers provided by the restaurant. We interviewed customers by telephone and face-to-face. Patient gastric samples and food samples were submitted for microbial testing. We conducted an environmental assessment. We performed Poisson logistic regression using R software.

**Results:**

Of 381 people identified, 364 were interviewed, of which 215 (59%) had become ill. Main symptoms were abdominal pain (98%), diarrhea (94%), nausea (94%), headache (91%), chills (82%), fever (81%), and vomiting (55%). Mean illness duration was 4 days (range=1-8). Mean onset time was 34 hours (range=6-91 hours) and 33% were hospitalized. Risk of illness was significantly higher among people who ate Caesar sushi rolls (1.55, 95% confidence interval [CI]=1.18-2.04), chicken and mushroom pizza (1.74, CI: 1.16-2.5), and "American" pizza (1.59, CI: 1.21-2.09). Among people who became ill, 60% had eaten Caesar sushi rolls and 40% american pizza. Attack rate was highest among those who had chicken pizza (97%). There was no common ingedient identified in the three foods. *E.coli* at concentration 10^3^ and *Enterococcus spp.* were detected in Caesar rolls. Of 22 gastric samples tested, *B. cereus* was detected in 45%, *Enterococcus* in 50%, *E. coli* in 23%. Environmental assessment found several food safety violations including improper storage conditions, prepared foods being stored with raw materials, and missing or expired workers' health and food certifications.

Factors associated with acute gastrointestinal illness, Talgar, Kazakhstan, 2023

**Figure:**
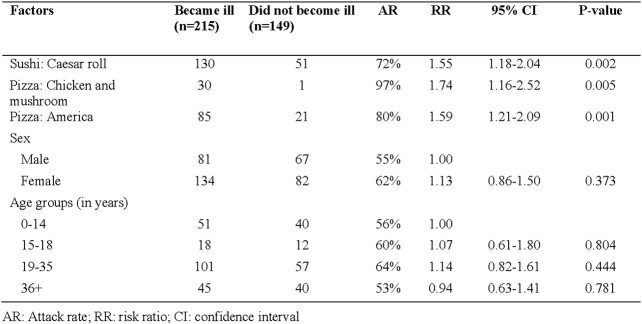

**Conclusion:**

Because multiple pathogens were detected in food and patient samples and disease was associated with multiple foods, we suspect that food contaminated at different points in the preparation process was likely the source of the outbreak. The restaurant was closed and sanitized. We made recommendations to improve food handling and safety practices.

**Disclosures:**

**All Authors**: No reported disclosures

